# Consent for use of personal information for health research: Do people with potentially stigmatizing health conditions and the general public differ in their opinions?

**DOI:** 10.1186/1472-6939-10-10

**Published:** 2009-07-24

**Authors:** Donald J Willison, Valerie Steeves, Cathy Charles, Lisa Schwartz, Jennifer Ranford, Gina Agarwal, Ji Cheng, Lehana Thabane

**Affiliations:** 1 Department of Clinical Epidemiology & Biostatistics, McMaster University, Hamilton, Ontario, Canada; 2 Surveillance and Epidemiology Division, Ontario Agency for Health Protection and Promotion, Toronto, Ontario, Canada; 3 Department of Criminology, University of Ottawa, Ottawa, Ontario, Canada; 4 Centre for Health Economics and Policy Analysis, Hamilton, Ontario, Canada; 5 Department of Philosophy, McMaster University, Hamilton, Ontario, Canada; 6 Department of Family Medicine, McMaster University, Hamilton, Ontario, Canada; 7 Biostatistics Unit, St Joseph's Healthcare, Hamilton, Ontario, Canada

## Abstract

**Background:**

Stigma refers to a distinguishing personal trait that is perceived as or actually is physically, socially, or psychologically disadvantageous. Little is known about the opinion of those who have more or less stigmatizing health conditions regarding the need for consent for use of their personal information for health research.

**Methods:**

We surveyed the opinions of people 18 years and older with seven health conditions. Participants were drawn from: physicians' offices and clinics in southern Ontario; and from a cross-Canada marketing panel of individuals with the target health conditions. For each of five research scenarios presented, respondents chose one of five consent choices: (1) no need for me to know; (2) notice with opt-out; (3) broad opt-in; (4) project-specific permission; and (5) this information should not be used. Consent choices were regressed onto: demographics; health condition; and attitude measures of privacy, disclosure concern, and the benefits of health research. We conducted focus groups to discuss possible reasons for observed consent choices.

**Results:**

We observed substantial variation in the control that people wish to have over use of their personal information for research. However, consent choice profiles were similar across health conditions, possibly due to sampling bias. Research involving profit or requiring linkage of health information with income, education, or occupation were associated with more restrictive consent choices. People were more willing to link their health information with biological samples than with information about their income, occupation, or education.

**Conclusions:**

The heterogeneity in consent choices suggests individuals should be offered some choice in use of their information for different types of health research, even if limited to selectively opting-out. Some of the implementation challenges could be designed into the interoperable electronic health record. However, many questions remain, including how best to capture the opinions of those who are more privacy sensitive.

## Background

The term "stigma" generally refers to a distinguishing personal trait that is perceived as or actually is physically, socially, or psychologically disadvantageous. [1] Because of the presence of that trait, an individual may be discriminated against – e.g. in employment or in social circles. Health conditions will vary in the extent to which they are perceived by those individuals having the condition and by others as being stigmatizing. Individuals with a potentially stigmatizing health condition may be more inclined than members of the public without such conditions to experience concerns over disclosure of their personal health information out of concern that this could result in discrimination against them. For example, a person with a prior history of cancer may be concerned over denial of certain employment opportunities, a mortgage, or life insurance. A person with HIV/AIDS may be concerned about social isolation because of others' concerns that their presence puts others at increased risk of contracting the condition.

There is now an emerging body of literature examining the opinion of the public regarding consent for use of one's health information for research. [2-9] However, the opinion of those who have health conditions that may be stigmatizing to a greater or lesser degree has been much less studied. [10] There are several reasons for considering – or even giving priority to – the values and expectations of these individuals. People who are unwell have the most at stake, both because they stand to benefit from research into their health condition and because a breach of privacy may potentially expose them to discrimination in obtaining loans, mortgages, insurance, or employment. Moreover, they are under-represented in surveys targeted to the general public and, to the extent that some health conditions are stigmatizing, the perspective of individuals with these conditions may be discounted by the general public. Further, if we listen only to the voice of the general public without attention to the concerns of this vulnerable minority we run the risk of committing a form of "tyranny of the majority". [11]

### Objective

The purpose of this study was to examine the attitudes of people with a range of potentially stigmatizing health conditions concerning the need for consent for the use of their personal information for different types of observational health research, and to compare their attitudes with those of the general public. We hypothesized that:

- responses would differ across health conditions;

- some patient groups would be more permissive and others more restrictive than the general public; and

- people's view of the level of consent required for use of their information would vary directly with disclosure concern and inversely with perceptions of the benefits of health care and the potential for health research to improve the lifespan and quality of life of people with their health condition.

This paper reports on the testing of these hypotheses.

## Methods

### Choice of health conditions

In this study, we included seven health conditions with varying susceptibility to being labelled as stigmatizing. Four conditions – hypertension, diabetes, chronic depression and alcoholism – were used in a previous public opinion survey of members of the public, in which respondents were asked to imagine they had one of these health conditions. [4] In the previous study, hypertension and diabetes were found to be lower-stigma health conditions. Chronic depression and alcoholism were found to be higher-stigma conditions. In this study, we had the opportunity to obtain the views of people with these conditions. To these four conditions we added: HIV, to create an upper extreme category for potential stigma; breast cancer; and lung cancer. We anticipated that responses may differ between breast and lung cancer because of a greater general public support for breast cancer sufferers and a perception that lung cancer is self-inflicted through smoking.

### Survey

The study proceeded in two phases. In Phase 1 (November 2006 to July 2007), we surveyed individuals with the target health conditions. In Phase 2 (July to September 2007), we held focus groups with a sub-sample of participants from Phase 1 – one group for each health condition. The chief purpose of Phase 2 was to help inform our analysis of the findings derived from Phase 1, by providing examples of the types of concerns some people took into account when making decisions around consent.

### Setting and Participants

Survey participants were drawn from two sources: (1) a pre-existing cross-Canada panel of individuals with identified health conditions, maintained by Harris Interactive, a professional polling firm; and (2) patients recruited by the investigators through family physicians' offices and specialty clinics in the vicinity of Hamilton, Ontario, Canada. The reference group consisted of people recruited through Harris Interactive who had none of the target health conditions and no other serious health conditions. This group was used to approximate the response of the general public. All survey participants were 18 years or older.

Participants recruited directly by the investigators were either sent a letter from the physician's office in the mail explaining the study or handed an information brochure in the clinic by staff. In each case, information was provided for patients to contact the investigators if interested. In total, 892 brochures were mailed to patients' homes and 888 brochures provided to physicians and clinics for directly handing out to patients.

All participants recruited through Harris Interactive completed the survey over the internet. Participants recruited through local family physicians and clinics were given the choice to complete the survey via internet or by telephone. This was done to minimize refusal due to lack of access to or familiarity with the internet, particularly among older patients. Those opting to do the internet survey used the same system as the Harris participants. Those opting to complete the survey by telephone arranged a scheduled call with telephone surveyors from Harris Interactive. They were mailed a hard copy of the core questions of the survey beforehand, so as to minimize differences in response due to method of survey administration.

Sample size was calculated on the basis of the primary outcome variable – consent choice in the use of personal information for health research. This was expressed on a 5-point ordinal scale. (See Survey Data and Key Variables below.) This sample was determined with the primary goal of building a multivariable regression model to compare the overall attitudes among the seven groups and the general public controlling for several demographic and other confounding variables. Heuristics based on simulation studies indicate that at least five respondents per degree of freedom for each predictor variable are required for the stability of the model. [12] We have 7 predictor variables with a total of 21 degrees of freedom, which would require at least 105 participants. We aimed to recruit 1400 and obtained responses from 1137 (734 from Harris Interactive and 403 from physician offices and clinics) with the sampling stratified by health condition. We inflated our minimum sample size by a factor of over 10 to account for potential clustering of responses within a patient. Therefore, the sample size was adequate to ensure the stability of the model.

### Survey Data and Key Variables

We collected information on participant demographics, attitudes about privacy, disclosure concern, and the benefits of health care and health research at improving longevity and quality of life, and the participant's health conditions. Where the participant had more than one target health condition, they were asked to answer the survey questions with only one health condition in mind. In earlier pilot work, we established an algorithm for determining which health condition would take priority, ranking the 7 health conditions according to level of disclosure concern. This algorithm placed HIV/AIDS first, followed by alcoholism, lung cancer, breast cancer, depression, diabetes and hypertension.

We presented five different scenarios involving use or linkage of personal information for health research: (1) use of health data for quality improvement; (2) use of the same data for marketing; (3) linkage of work/education/income information with health information; (4) linkage of biosamples with health information (a) assuming no profit and (b) assuming a profit element. (See additional file 1 for a more detailed description.) These scenarios were identical to those used two years earlier in a series of seven cross-Canada public dialogues. [13] Participants of the current study were advised that, in each scenario, names, addresses and any other information that could directly identify them were removed. Following each scenario, participants were asked which statement best described their view (words in italics not included in survey responses):

(1) There is no need for me to know. Just use it.

(2) My permission is not needed, but I want to know this is being done and a chance to say "no." *[i.e. notice with opt-out]*

(3) My general permission is needed. This could be for several different research studies. I could withdraw my permission in future. *[i.e. broad opt-in]*

(4) My permission is needed each time. *[i.e. project-specific consent]*

(5) My information should not be used for this purpose.

The five response options above, hereinafter referred to as "consent choices", served as the outcome variable in a multivariable regression analysis using the following as predictor variables:

- demographics (age, sex, education, marital status, employment, and income)

- health condition (one of the 7 target conditions)

- self-reported health (6-point scale, varying from "very poor" to "excellent")

- scenario, and

- attitudinal variables (disclosure concern and medical benefits score. These are described below. Questions used to compile these scores and the scoring scheme are found in additional file 2.)

While there are many dimensions to stigma, for the purposes of this study, we chose to focus on individuals' disclosure concern – i.e. concern that others may find out about their health condition. For this, we asked: How concerned would you be if: (a) your employer found out about any health condition(s) you have; (b) your health insurer found out about any health condition(s) you have; or (c) a friend other than those you told found out about any health conditions you have. For each of these questions, respondents replied either: "not at all concerned"; "somewhat concerned"; or "very concerned".

For the medical benefits scale, people replied on a 5-point scale ("strongly agree, somewhat agree", "neither agree nor disagree", "somewhat disagree", or "strongly disagree") to the following statements: (a) Medical treatments can improve the quality of my life; (b) medical treatments can extend my life; (c) disease prevention programs have shown me how to live a healthier life; and (d) medical research can improve my life.

We re-scaled the disclosure concern and medical benefits scores to a 0–1 scale to facilitate interpretation of relative attitudes toward disclosure and medical benefit across health conditions.

### Dealing with potential sampling bias

We checked for sampling bias chiefly through two methods. Harris Interactive sampled questions from our survey in an omnibus random-digit dialled telephone survey and compared the responses from the telephone survey with those from their internet sample. In addition, we compared the consent choices of the reference group from this study regarding the five scenarios with the consent choices of the people who participated in the public dialogues in our previous study. [13]

### Statistical methods

Consent choices were analysed graphically across scenarios and across health conditions. We also plotted disclosure concern and medical benefit scores across health conditions using a radar graph.

To test our chief hypotheses, we used regression analysis controlling the correlation across scenarios using the method of generalized estimating equations (GEE) assuming an exchangeable correlation structure. [14] The results are reported as estimates of model coefficients (with corresponding 95% confidence interval) and associated p-value. Statistical computations used SAS, version 9.1.3 (Cary, NC.). In earlier work, we found the results of linear regression to yield equivalent results to multinomial logistic regression which is the more correct analysis but more difficult to interpret. [4]

Initially, individual predictor variables were regressed onto consent choice using univarite analysis. Variables that met the criterion of alpha = 0.20 were then included in the multivariable regression model. The chief variables of interest – health condition, disclosure concern, and medical benefit scores – were forced into the model. We tested for interaction effects of disclosure concern and medical benefits scores with research scenarios and health condition, No significant interactions were found.

### Focus Groups

At the end of the survey, those participants recruited directly by the investigators in the Hamilton area were asked if they would be willing to participate in a focus group to discuss the reasoning that participants may have used to make their consent choices. Those who agreed were contacted by the study coordinator. One focus group was convened for each health condition except for alcoholism, as the survey sample size was too small for this group. We sampled from the pool of survey participants to provide a representative sample in each focus group with regard to age, gender, and self-reported health status. When making selections, investigators were blind as to volunteers' responses to survey questions.

Six focus groups, ranging in size from 6 to 10 individuals, were conducted between July and September 2007. Focus groups were 90 minutes in length. Participants received an honorarium of $75 following the session. These meetings were moderated by two of the researchers. Upon arrival, participants completed a mini-survey comprised of the key questions from the Phase 1 survey. During the focus group, a structured interview protocol was used to ask participants to reflect on the survey findings regarding consent choice for the five different research scenarios. Participants were then asked to consider how the survey responses compared with their own responses and to consider the reasons for their responses and any possible variance from the response pattern from the Phase 1 survey. Focus group participants were also asked to comment on the relative importance of individual control over use of their information and safeguards and the relation between them. This question was addressed both in the abstract and by giving them the opportunity to rate specific controls and safeguards. Immediately following the meeting, participants again completed the mini-survey, so a comparison of the responses of focus group participants with those of the larger sample could be made. Discussions were audio recorded and transcribed for analysis.

Verbatim transcripts from all focus groups were read independently by at least two members of the research team and quotations were selected to exemplify the types of reasoning that focus group participants used to make their consent choices. These quotations are used below to help illustrate the broader survey analysis, and to provide a window on the kinds of issues some people may address when making consent choices.

### Ethics Review

The research was reviewed and approved by the research ethics boards of St. Joseph's Healthcare, Hamilton, Ontario, McMaster University Health Sciences REB, Hamilton, Ontario and the Social Sciences and Humanities REB, University of Ottawa, Ottawa, Ontario.

## Results

### Participants

**Figure 1:**
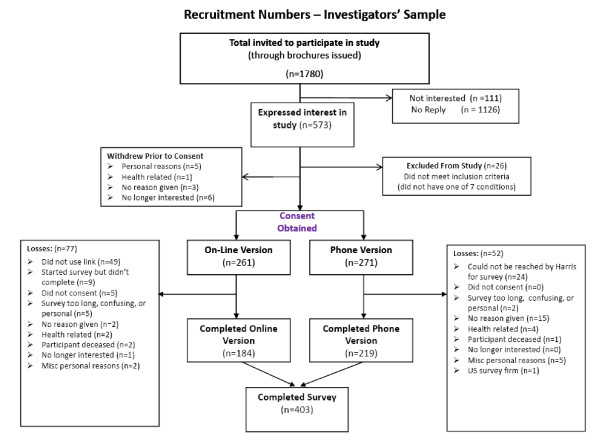
**Summary of recruitment process, using the investigators' sample**.

Four hundred three survey participants were recruited directly by the investigators and 734 were recruited through Harris Interactive. Recruitment of survey participants through the investigators' sources is summarized in Figure 1.

### Demographic data

Additional file 3 summarizes the demographic characteristics of study participants by sampling source. Those recruited directly by the investigators are presented by method of survey completion. Overall, the reference group was younger (mean age 46 years) than participants with target health conditions (mean 55 years), regardless of sampling source. Their self-reported health was better than those with the target health conditions. Those with the target health conditions completing the survey over the internet were comparable between the two sampling sources on most demographic variables. However, the Harris sample was less wealthy and reported poorer health than the sample assembled by the investigators. Survey participants recruited by the investigators who completed the survey by telephone were older (mean age 62) with a greater percentage of women (68% vs. 60% in general public and 53% and 57% in the Harris and investigator internet samples). Telephone survey participants had a greater percentage with high school education or less (56%) than those who completed the survey over the internet (28%). In addition, telephone respondents were more likely to be separated, widowed, or divorced (30% vs. 14–24%). Fewer were employed (21% vs. 49–72%) and a greater percent had an income less than $40,000 per year (42% vs. 20–35%).

### Attitudes

**Figure 2:**
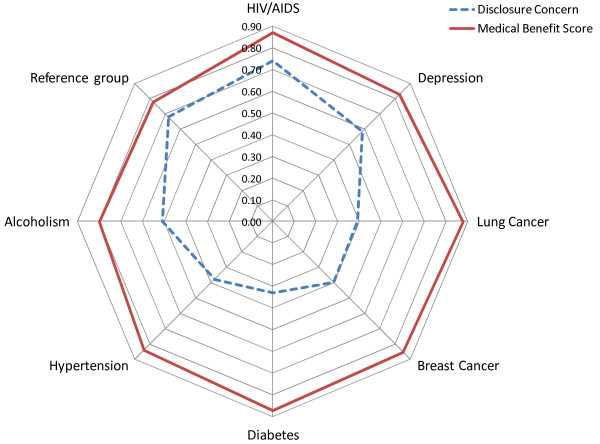
**Attitudes of survey participants across health conditions**.

A summary score of participants' perceptions about the benefits to be accrued from health care and health research (medical benefits score) and disclosure concern is provided in Figure 2. Each axis on this radar plot represents one health condition or the reference group. The scale on the axis for both attitudes was standardized to (0,1) to facilitate comparison. The medical benefits score was uniformly high across health conditions. On the other hand, there was substantial variation across the health conditions regarding the level of concern participants had about employers, insurers, or friends finding out about their health condition. Disclosure concern was lowest for those with hypertension, diabetes, and lung cancer. It was highest among those with chronic depression and HIV/AIDS. We note also that disclosure concern in the reference group was almost as high as the HIV/AIDS group yet, they had no serious or chronic health condition.

### Consent choice

**Figure 3:**
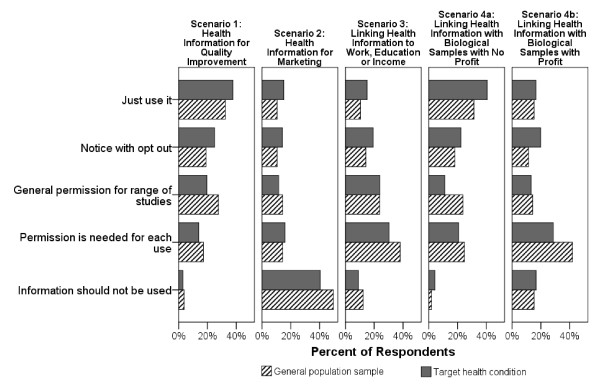
**Consent choices across research scenarios – A comparison of the general population with a pooled sample of those with the target health conditions**.

Across scenarios, consent choice profiles were very similar for all health conditions. They were also very similar to the profile of the reference group. For ease of presentation, we have combined the responses across health conditions in Figure 3 and compared these with the reference group.

#### Effect of intended use and commercialization on consent choice

Scenario 1 involved the use of prescribing information from the medical record for quality improvement and, for this paper, serves as the comparator scenario. Figure 3 shows a relatively permissive consent profile for this scenario, with the most common response being "Just use it" (35–40%). By contrast Scenario 2, which used the same prescribing information for marketing purposes, elicited essentially the opposite profile, with over 40% of respondents indicating that this information should not be used (at all) for this purpose.

A similar, though less marked, shift in the pattern of consent choice was observed when comparing Scenarios 4a and 4b. In Scenario 4a, individuals' health information was linked with biological samples in the absence of any commercialization of any discovery. In this case, the consent profile was quite similar to that for Scenario 1. When a potential profit element was introduced through the development of a commercial lab test (Scenario 4b), the consent profile displayed a desire for greater control over use of that information, though not as strong as for the marketing scenario (Scenario 2), and with no particular consent choice being preferred.

In our focus groups, we probed for explanations behind the desire for greater control when commercialization was involved. The responses were wide ranging. A common sentiment was that people felt they were being taken advantage of:

*"It was a matter of control. It's the whole idea of profit, that word 'profit'. Once I see that, I just have a sense of being taken advantage of. ... but on the other hand I wouldn't not do it because it is helpful. I would just want to know." *(Participant 7, Diabetes group)

*"First thing I thought of was 'Well, if they're selling it for a profit what do I get out this?' I just don't see your volunteering something, if somebody else is making a profit out of it. I don't see that." *(Participant 3, Lung cancer group)

#### Different consent profiles for linkage with biological samples vs. income, education, and occupation, in the absence of profit

The consent profile for linkage of health information with biological samples in the absence of profit (Scenario 4a) was similar to that for the quality improvement scenario (Scenario 1). Thirty to 40% of respondents felt it was acceptable to link this information without notification. By contrast, linkage of health information with income, education, or occupation (Scenario 3) was associated with a consent profile that reflected a desire for greater control over use of the information. For this scenario, only 11–15% felt it was acceptable to link this information without notification and 30–43% of respondents felt their consent should be sought for each use before the information could be linked.

Because this finding surprised us, we probed this in our focus groups. Similar sentiments were expressed across groups, around 3 categories of explanations:

1. Participants felt that information about income, education or occupation says a lot about "who we are" whereas a biosample only provides information about "what we are".

*"I think the simple answer is that physical tissue sample is just a piece of what you are, what you might be...where the rest of the information [education, income, and employment] is more of who you are. People are more afraid of the revelation of who you are than what you are." *(Participant 8, HIV group)

2. Participants believed that access to information about education, income, and occupation can lead to the identification of the individual, but biosamples cannot.

*"The work, education and your income. If somebody looks at a biological sample they can't look up and say 'That's you or you.' You can identify somebody by all that, those other things." (*Participant 6, Depression group)

*"The only thing you can get from tissue or bodily fluids is DNA and what the disease is. They can't get information about you per say. Right?" (*Participant 3, Lung cancer group)

3. Participants trusted that their doctor and the medical system will ensure that the information will be used appropriately.

*"I think with tissue you can see a real concrete connection. You can imagine a scientist in a lab studying that tissue, looking for an answer. With income and work you're not sure what types of people are analyzing that. It's not a white lab coat, microscope it's more of a psychological marketing." (*Participant 7, Depression)

*"I trust giving out the information to most of the medical profession but it's not something I would give willingly to people that I don't really know well." *(Participant 3, Depression)

*"I have found when I've been asked to [do] this that my family Doctor, where I go – and I have trust in him and in his staff – and he sent a letter saying 'This is what they are asking you to do and I believe in it.' And so I feel that gave me the confidence that this wasn't just some little thing that showed up at my door. My family doctor... I have trust in him." (*Participant 8, Diabetes)

**Figure 4:**
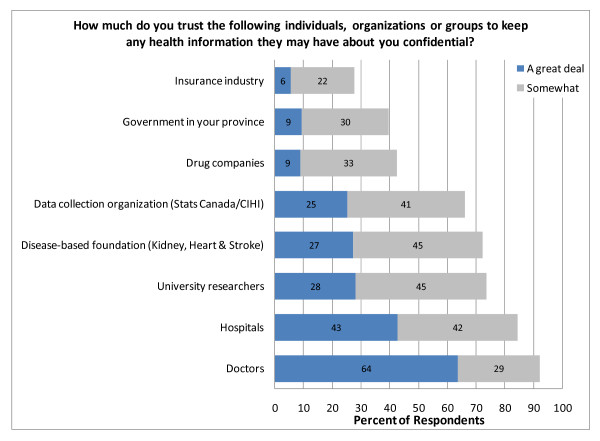
**Trust in organizations**.

These (and other) references to high trust were directed more toward doctors and hospitals or, even more personally, to *their *doctor. While there was also a relatively high trust toward university researchers (Figure 4), that trust was more tentative than for doctors.

### Regression analyses

Additional file 4 compares three regression models. The full model examines the independent contributions of health condition and attitudes toward disclosure concern and medical benefits, controlling for scenario, survey method, and sex. Reduced model 1 removes disclosure concern and medical benefit from the model while reduced model 2 removes health condition from the model.

In the full model, consent choices among survey participants did not differ significantly across health conditions, compared with the reference group. Increased disclosure concern was associated with a more restrictive consent choice while greater perception of the benefits of medical care and medical research was associated with more permissive consent choice. When the attitude variables (disclosure concern and medical benefits scores) were removed from the model (Reduced Model 1), the coefficients for several of the health conditions moved in the direction of being more permissive compared with the reference group. In two cases (breast cancer and hypertension) these became statistically significant. On the other hand, when health conditions were removed from the model (Reduced Model 2), estimates for the attitude variables were s . This suggests that the stronger predictor of consent choice was individual attitude toward medical benefit and disclosure concern than was the person's health condition.

Regression results also confirm our perceptions that research involving an element of profit (Scenarios 2 and 4b) or requiring linkage of health information with income, education, or occupation (Scenario 3) were associated with more restrictive consent choices. Female respondents were also generally more restrictive in their consent choices. Those completing the survey by telephone were more permissive in their consent choices than were those completing the survey over the internet.

## Discussion

### Chief findings

We had hypothesized that consent choice would differ across the selected health conditions and also with individual attitudes regarding disclosure concern and perceptions of the benefits of medical care and research. While we recognized beforehand that hypertension and diabetes were relatively low in stigma and that HIV and alcoholism were likely higher in stigma, we did not assume strict ordinality across health conditions. We found that, collectively, participants with target health conditions were slightly more permissive in their consent choices compared against the reference group (Figure 3). However, when we controlled for survey method, sex, and attitudes, we found no statistically significant difference in consent choice across health conditions, compared with our reference group – this despite having chosen health conditions that differed widely in potential for stigma. When examining the confidence intervals around the parameter estimates for the health conditions, they did not appear to be of a magnitude that would be policy relevant. These findings surprised us. It may be that, across health conditions, those who were more privacy sensitive were less inclined to participate in the study and those were more permissive about use of their information were more inclined to participate. This is discussed further under "Limitations" below.

Disclosure concern differed across health conditions and was associated with more restrictive consent choices. By contrast, there was a uniformly high rating of medical benefits across health conditions. However, at the individual level, a lower perception of medical benefits was associated with more restrictive consent choices. Thus, individual attitudes – and disclosure concern in particular – were more predictive of consent choice than was one's health condition. This suggests that privacy attitudes may be formulated relatively early on and may be robust to one's health condition, which may develop later in life.

We also observed significantly more restrictive consent choices in research scenarios involving profit or when linking health information with income, education, or occupation. This is consistent with findings from our earlier public dialogues. [13] We also note that people were relatively trusting with regard to linking their health records to their biological samples – even more trusting than with linking to income, education, and occupation. This is an interesting finding worthy of further study, given that there is a much higher likelihood of commercial application and intellectual property protection in research involving biological samples and the great potential for stigmatization and discrimination. Our focus groups suggest that, at least in part, their lesser concern was attributable to perceptions that specialized knowledge was required to interpret one's DNA and those with that specialized knowledge were trusted to keep the information confidential. However, once one's genetic risk profile is recorded in the health record, this information is equally subject to misadventure as one's income, should that information fall into the wrong hands. With regard to trust, we note that most of the focus group discussion of trust was in reference to their doctors. Perhaps trust in one's doctor then confers benefits of trust to the researchers in the process.

### Limitations

Based on a comparison with a subset of questions in an omnibus survey conducted by Harris Interactive, we determined that people recruited into the study were somewhat less privacy concerned and more research-friendly than the general public. Within our study sample, those who completed the survey by telephone were particularly less privacy concerned. This observation is reinforced by comparison of our current findings with those of our earlier study involving a cross-Canada sample of the general public using random-digit dialling. In the earlier study, the profiling of consent choices was somewhat less permissive than observed here – particularly around the linkage with genetic information. [13] Intuitively, one would expect that individuals willing to participate in ongoing internet consumer panels may be less privacy concerned and more open to participating in research. Our conventional sampling through clinics, though, was also subject to a similar selection bias. Current ethics rules require that a researcher not approach potential research participants directly, based on prior knowledge about that individual's health condition. Individuals must first be asked by someone who may be reasonably expected to have access to this information if they would be willing to have their name released so they could be called by the researcher, or if they would be willing to take the initiative themselves to contact the researcher. This two-stage process probably resulted in lack of access to more privacy-sensitive research participants.

The reference group in this study is not quite representative of the "general public", to the extent that: (a) they, too, were drawn from the Harris internet polling sampling frame; and (b) they consisted of individuals who did not have any of the target health conditions and who had no other major health conditions. A true random sample of the public would have some proportion of respondents with the target health conditions.

Given all this, we believe our study may have under-represented those in our society who are the most privacy sensitive. Moreover, depending on the severity of the selection bias, it may be that our failure to observe a difference in consent choice across health conditions may be a result of the absence of more privacy-sensitive respondents. These non-participants may represent varying proportions across health conditions. We cannot assess what proportion of the selection bias is due to self-selection or selective approaching by their physicians.

Finally, in the consumer literature, stated privacy preferences are often much more stringent than those revealed in actual behaviour. [15] Thus, it is possible that the stated consent choices for use of one's personal information reported here may be different than what they are prepared to accept in the health care "marketplace".

### Policy implications

No one consent option was preferred by even a simple majority of survey participants in any of the scenarios (Figure 3). This wide variation in opinion is consistent with our earlier work with the general public and with a recent American survey. [3] This high heterogeneity in consent choices makes it difficult to put forward any one model for consent for research use of one's personal health information. One possible response would be to offer individuals a menu of choices with a mechanism to track their choices re: secondary uses of data. While this is now technically possible, there are multiple challenges from a systems perspective. For example, who would broker the consent process and under what conditions? Anecdotal evidence suggests that physicians do not have the time for this. On the other hand, special "clinics" could be set up through hospitals and centres where applications are made for renewal of health cards. Information could be available through brochures and DVDs, and knowledgeable individuals could be available either in person or over the telephone. All these approaches would require a substantial investment in funds and an ongoing infrastructure for managing these consent choices. This could also be designed into the planned pan-Canadian interoperable electronic health record system, through secure web-based patient portals into their health record. [16,17] These portals are web-based interactive systems that allow individuals to view their health record and communicate in a secure fashion with their health care provider's office. Another consideration is whether consent choices should be totally unconstrained. There is growing evidence that opt-in consent processes can result in selection biases that may affect conclusions as to various causal associations. [18] Are there certain types of research (e.g. public health, quality of care) for which the default assumption would be that the information may be used for research and people's only option would be to selectively opt out? These are but two examples of issues that need to be addressed through further research.

Our survey and focus group participants placed high trust in medical researchers – higher than that found recently by Westin in the United States. [3] However, as in Westin's study, much of this trust was qualified. Approximately two-thirds of those expressing trust in university researchers only "somewhat trusted" them. Therefore, much of this trust in researchers is vulnerable to erosion in the event of a high-profile breach of confidence. Survey participants also valued highly the ability to monitor how their information was being used and the ability to say "No" to particular uses. This "trust but verify" attitude could also be accommodated through the development of a patient portal into one's electronic health record.

We observed a shift toward more restrictive consent choices when research involved a commercial element. Given the increasing importance of personal information to health research, and the pervasiveness of the private sector and commercialization in the research enterprise, continued public engagement would be worthwhile. This would help researchers to better appreciate the underlying concerns of the public around research and profit and to determine whether these views change or are reinforced with a better understanding of the role of both personal information and the private sector in research.

## Conclusions

The public is generally supportive of research use of personal health information, but do not wish to entirely relinquish control. Despite having a relatively research-friendly sample, we still observed substantial individual variation in opinion as to the degree of control that people wish to have over use of their personal information for research. Opportunities for individual choice over use of one's personal health information could be designed into the planned pan-Canadian inter-operable electronic health record system.

People's desire for control over use of their personal health information increases when there is a commercial element to the research. Additional public engagement is required to better understand this.

Finally, this study attempted to address the question of how much control patients would like over use of their personal health information for research. While we fully appreciate the rationale for restricting researchers from directly recruiting patients on the basis of prior knowledge about their health condition, it is ironic that, to meet ethical requirements respecting individuals' privacy, this study has likely under-represented the interests of those very people whose voices need to be heard. When addressing questions like the one we have posed here, there need to be ways to better reach those who have the most at stake.

## Competing interests

The authors declare that they have no competing interests.

## Authors' contributions

DW conceived and designed the study, led the focus groups and the analyses and was the primary author of the manuscript; VS, CC, LS, JR and LT all contributed to the development of the protocol. JR led in the analyses of the text. LT provided statistical guidance. JC conducted the statistical analyses. All co-authors reviewed and revised drafts of the manuscript. All authors read and approved the final manuscript.

## Pre-publication history

The pre-publication history for this paper can be accessed here:



## Supplementary Material

Additional file 1 **Box 1 – Description of Research Scenarios**. Provides information about the scenarios on which consent choices were made. Click here for file

Additional file 2 **Appendix 1. Survey questions that formed the elements for (a) the disclosure concern scale; (b) the medical benefits scale; and (c) the consent choices outcome variable**. This document provides the core questions that were used in developing two key predictor variables (a measure of individuals' concern over disclosure of information about their health condition and a measure of individuals' perceptions of the benefits of health care and health research for their health condition) and the key outcome variable (consent choice). Click here for file

Additional file 3 **Table 1. Summary of Participant Demographics**. This describes basic demographic features of survey participants broken down by survey method and sample source. We divided investigator sample source into those who responded by telephone and internet to determine whether demographic characteristics varied more by sample source or self-selected method of completion of the survey. Click here for file

Additional file 4 **Table 2. Regression analysis of consent choice predictors**. This table presents the results of regression modelling of predictors of consent choice – scenario, survey method, health condition, sex, disclosure concern score, and medical benefits score. The latter two variables were a composite of several questions in the survey. (See Additional File 2.) The comparison of reduced models 1 and 2 allows one to compare the relative amount of explained variance in consent choices that would be attributable to (a) health condition and (b) the combination of perceptions of medical benefit and disclosure concern. Click here for file
