# Supplementary material for: Consent for use of personal information for health research: Do people with potentially stigmatizing health conditions and the general public differ in their opinions?
Source: BMC Med Ethics. 2009 Jul 24;10:10. doi: 10.1186/1472-6939-10-10 (PMC2724473; doi:10.1186/1472-6939-10-10)
Supplement: Additional file 1 — Box 1 – Description of Research Scenarios. Provides information about the scenarios on which consent choices were made. [file 1472-6939-10-10-S1.doc]

| **Scenario synopsis** | | **Scenario description** |
| --- | --- | --- |
| Scenario 1 – Health Information for Quality Improvement | | Research that tracks how doctors prescribe medications, to give them feedback to help them improve the care they provide. |
| Scenario 2 – Health Information for Marketing | | Research that tracks how doctors prescribe medications so drug companies can better target their advertising to doctors. |
| Scenario 3 – Linking Health Information to Work, Education or Income | | Research that looks at the relationship between health and work, education or income. To do this research, information about your work, education or income must be combined with information from your health record. |
| Scenario 4 –Linking Health Information with Biological Samples | | Research that studies leftover tissue or fluid following surgery to better understand the cause or treatment of the disease. To do this, your age, sex, diagnosis and other medical conditions would be linked with the sample. |
|  | 4a With No Profit | Please assume that the researchers have no plans to develop a commercial product, like a lab test, that is sold for profit. |
|  | 4b – With Profit | If the goal of the research is to identify a new test that could better diagnose if you had a condition that needed the surgery. The lab test would be sold for profit. |
