# Supplementary material for: Consent for use of personal information for health research: Do people with potentially stigmatizing health conditions and the general public differ in their opinions?
Source: BMC Med Ethics. 2009 Jul 24;10:10. doi: 10.1186/1472-6939-10-10 (PMC2724473; doi:10.1186/1472-6939-10-10)
Supplement: Additional file 2 — Appendix 1. Survey questions that formed the elements for (a) the disclosure concern scale; (b) the medical benefits scale; and (c) the consent choices outcome variable. This document provides the core questions that were used in developing two key predictor variables (a measure of individuals' concern over disclosure of information about their health condition and a measure of individuals' perceptions of the benefits of health care and health research for their health condition) and the key outcome variable (consent choice). [file 1472-6939-10-10-S2.doc]

**Questions forming the foundation of the Disclosure Concern scale**

How concerned would you be if your employer found out about any health condition(s) you have?

- Not at all concerned (score = 0)
- Not very concerned (score = 1)
- Somewhat concerned (score = 2)
- Very concerned (score = 3)
- Does not apply

How concerned would you be if your health insurer found out about any health condition(s) you have?

- Not at all concerned (score = 0)
- Not very concerned (score = 1)
- Somewhat concerned (score = 2)
- Very concerned (score = 3)
- Does not apply

How concerned would you be if a friend other than those <U>you</U> told found out about any health conditions you have?

- Not at all concerned (score = 0)
- Not very concerned (score = 1)
- Somewhat concerned (score = 2)
- Very concerned (score = 3)
- Does not apply

For the disclosure concern score, some participants answered 3 questions while some answered 2 questions only, as those who were retired may have replied ‘Does not apply’. Further a very few indicated 2 of the questions did not apply, so answered only one question. The original scale from “not at all concerned’ to “very concerned’ is from 0 to 3 for 3 related questions. To create (0,1) score, for those participants who answered 3 questions, the raw score was divided by 9; for those who answered 2 questions, the raw score was divided by 6; and for those who only answered 1 question, the raw score was divided by 3.

**Questions forming the foundation of the Medical Benefits scale**

For each of the statements below, please choose the response that most closely matches your opinion.

Medical treatments can improve the quality of my life.

❑ strongly agree (score = -2)

❑ somewhat agree (score = -1)

❑ neither agree nor disagree (score = 0)

❑ somewhat disagree (score = 1)

❑ strongly disagree (score = 2)

Medical treatments can extend my life.

❑ strongly agree (score = -2)

❑ somewhat agree (score = -1)

❑ neither agree nor disagree (score = 0)

❑ somewhat disagree (score = 1)

❑ strongly disagree (score = 2)

Disease prevention programs have shown me how to live a healthier life.

❑ strongly agree (score = -2)

❑ somewhat agree (score = -1)

❑ neither agree nor disagree (score = 0)

❑ somewhat disagree (score = 1)

❑ strongly disagree (score = 2)

Medical research can improve my life.

❑ strongly agree (score = -2)

❑ somewhat agree (score = -1)

❑ neither agree nor disagree (score = 0)

❑ somewhat disagree (score = 1)

❑ strongly disagree (score = 2)

**Scale development:** For the medical benefit score, the original scale was from -2 to 2 for 4 related questions. When summed across the four questions, the total raw score was from -8 to 8.  The scale was standardized to a (0, 1) range by: (a) shifting the original (-2,2) scale for each question to (0,4), thus creating a raw score ranging from 0 to 16. To create the (0, 1) score, the total score (min=0, max=16) was divided by 16.

**Sample scenario for determining Consent Choices – Scenario 3**

Please select the answer that best matches your opinions about the use of your information for each kind of research. To answer, please enter the number of the response for each statement in the box provided.

Research that looks at the relationship between health and work, education or income. To do this research, information about your work, education or income must be combined with information from your health record.

| 1. This information should not be used for this purpose. | (score = 4) |
| --- | --- |
| 1. My permission is needed each time. | (score = 3) |
| 1. My general permission is needed. This could be for several different research studies. I could withdraw my permission in future. | (score = 2) |
| 1. My permission is not needed, but I want to know this is being done and a chance to say “No” | (score = 1) |
| 1. There is no need for me to know. Just use it. | (score = 0) |
