# Supplementary material for: Consent for use of personal information for health research: Do people with potentially stigmatizing health conditions and the general public differ in their opinions?
Source: BMC Med Ethics. 2009 Jul 24;10:10. doi: 10.1186/1472-6939-10-10 (PMC2724473; doi:10.1186/1472-6939-10-10)
Supplement: Additional file 3 — Table 1. Summary of Participant Demographics. This describes basic demographic features of survey participants broken down by survey method and sample source. We divided investigator sample source into those who responded by telephone and internet to determine whether demographic characteristics varied more by sample source or self-selected method of completion of the survey. [file 1472-6939-10-10-S3.doc]

| **Variable** | **Category** | **Sample overall** | **Reference Group** | **Samples with Target Health Conditions** | | |
| --- | --- | --- | --- | --- | --- | --- |
|  |  |  | Harris internet | Harris internet | Investigator internet | Investigator telephone |
| Sample size |  |  | (n=105) | (n=629) | (n=184) | (n=219) |
| Age | Mean age (years) | 54 | 46 | 53 | 52 | 62 |
| Sex (%) | Female | 57 | 60 | 53 | 57 | 68 |
| Education (%) | High school or less | 33 | 24 | 28 | 28 | 56 |
| Marital status (%) | Single | 13 | 14 | 15 | 10 | 8 |
|  | Married / Common-law | 64 | 68 | 61 | 76 | 62 |
|  | Separated/Widow/Divorced | 23 | 18 | 24 | 14 | 30 |
| Employment (%) | Employed FT or PT | 47 | 72 | 52 | 49 | 21 |
| Income (%) | < $40,000 | 33 | 23 | 35 | 20 | 42 |
|  | $40,000 - $59,999 | 18 | 22 | 16 | 16 | 21 |
|  | $60,000 - $79,999 | 12 | 19 | 12 | 14 | 8 |
|  | $80,000 + | 24 | 21 | 25 | 38 | 11 |
|  | Refused / Don’t know | 14 | 15 | 12 | 13 | 19 |
| Self-described health (%) | Poor or very poor | 33 | 17 | 37 | 28 | 34 |
|  | Fair | 40 | 41 | 41 | 37 | 37 |
|  | Good or excellent | 27 | 43 | 22 | 35 | 28 |
