# Supplementary material for: Consent for use of personal information for health research: Do people with potentially stigmatizing health conditions and the general public differ in their opinions?
Source: BMC Med Ethics. 2009 Jul 24;10:10. doi: 10.1186/1472-6939-10-10 (PMC2724473; doi:10.1186/1472-6939-10-10)
Supplement: Additional file 4 — Table 2. Regression analysis of consent choice predictors. This table presents the results of regression modelling of predictors of consent choice – scenario, survey method, health condition, sex, disclosure concern score, and medical benefits score. The latter two variables were a composite of several questions in the survey. (See Additional File 2.) The comparison of reduced models 1 and 2 allows one to compare the relative amount of explained variance in consent choices that would be attributable to (a) health condition and (b) the combination of perceptions of medical benefit and disclosure concern. [file 1472-6939-10-10-S4.doc]

|  | **Full Model** (n=5402) | | | **Reduced Model 1** (n=5556) | | | **Reduced Model 2** (n=5402) | | |
| --- | --- | --- | --- | --- | --- | --- | --- | --- | --- |
|  | β^*^ | 95% C.I. | P-value | β^*^ | 95% C.I. | P-value | β^*^ | 95% C.I. | P-value |
| **Scenario** (Reference = Health Information for Quality Improvement) |  |  |  |  |  |  |  |  |  |
| Health Information for Marketing | 1.35 | (1.25, 1.45) | <0.001 | 1.35 | (1.25, 1.45) | <0.001 | 1.35 | (1.25, 1.45) | <0.001 |
| Linking Health Information to Work, Education or Income | 0.80 | (0.73, 0.88) | <0.001 | 0.80 | (0.73, 0.88) | <0.001 | 0.80 | (0.73, 0.88) | <0.001 |
| Linking Health Information with Biosamples – no profit | 0.06 | (-0.01, 0.13) | 0.091 | 0.07 | (-0.002, 0.13) | 0.056 | 0.06 | (-0.01, 0.13) | 0.091 |
| Linking Health Information with Biosamples with Profit | 0.91 | (0.83, 0.99) | <0.001 | 0.90 | (0.82, 0.98) | <0.001 | 0.91 | (0.83, 0.99) | <0.001 |
| **Survey Method** = telephone (Reference = Internet) | -0.31 | (-0.46, -0.16) | <0.001 | -0.34 | (0.49, 0.19) | <0.001 | -0.34 | (-0.49, -0.19) | <0.001 |
| **Health Condition**  (Reference = Harris control) |  |  |  |  |  |  |  |  |  |
| HIV/AIDS | 0.10 | (-0.20, 0.40) | 0.502 | 0.12 | (-0.19, 0.42) | 0.456 |  |  |  |
| Alcoholism | -0.02 | (-0.24, 0.20) | 0.869 | -0.10 | (-0.32, 0.12) | 0.385 |  |  |  |
| Lung Cancer | -0.21 | (-0.60, 0.19) | 0.303 | -0.35 | (-0.73, 0.03) | 0.069 |  |  |  |
| Breast Cancer | -0.10 | (-0.33, 0.14) | 0.426 | -0.23 | (-0.46, 0.00) | 0.050 |  |  |  |
| Depression | -0.13 | (-0.35, 0.08) | 0.224 | -0.18 | (-0.39, 0.03) | 0.097 |  |  |  |
| Diabetes | 0.08 | (-0.15, 0.29) | 0.528 | -0.12 | (-0.34, 0.09) | 0.247 |  |  |  |
| Hypertension | -0.13 | (-0.36, 0.10) | 0.262 | -0.28 | (-0.50, -0.06) | 0.013 |  |  |  |
| **Sex** (reference = male) Female | 0.13 | (0.01, 0.25) | 0.030 | 0.13 | (0.01, 0.25) | 0.036 | 0.10 | (-0.01, 0.21) | 0.074 |
| **Disclosure Concern Score** | 0.42 | (0.23, 0.61) | <0.001 |  |  |  | 0.42 | (0.24, 0.59) | <0.001 |
| **Medical Benefit Score** | -0.60 | (-0.91, -0.28) | <0.001 |  |  |  | -0.58 | (-0.89, -0.27) | <0.001 |
|  |  |  |  |  |  |  |  | | |
| Full model: chi-sq=5402,degree of freedom=5386, p-value of goodness of fit=0.436; intra-class correlation coefficient=0.363, R-square=0.169 | | | | | | | | | |
| Reduced model1: chi-sq=5556, degree of freedom=5542, p-value of goodness of fit=0.445; intra-class correlation coefficient=0.380, R-square=0.153 | | | | | | | | | |
| Reduced model2: chi-sq=5402, degree of freedom=5393, p-value of goodness of fit=0.463; intra-class correlation coefficient=0.367, R-square=0.165 | | | | | | | | | |
| Comparison of R-square: From reduced model 1 to full model, R-square increased 10.94%; from reduced model 2 to full model, R-square increased 2.36%. | | | | | | | | | |

^*^  A positive beta-coefficient indicates a more restrictive consent choice whereas a negative coefficient represents a more permissive choice
